# Micronization potentiates curcumin’s anti-seizure effect and brings an important advance in epilepsy treatment

**DOI:** 10.1038/s41598-018-20897-x

**Published:** 2018-02-08

**Authors:** Kanandra Taisa Bertoncello, Gean Pablo S. Aguiar, J. Vladimir Oliveira, Anna Maria Siebel

**Affiliations:** 1 Laboratory of Genetics and Molecular Ecotoxicology, Programa de Pós-Graduação em Ciências Ambientais, Universidade Comunitária da Região de Chapecó, Chapecó, SC Brazil; 2 Department of Chemical and Food Engineering, Universidade Federal de Santa Catarina, Florianópolis, SC Brazil

## Abstract

Epilepsy is one of the most common neurological diseases, and current antiepileptic drugs fail to suppress seizure occurrence in around one third of epileptic patients. Curcumin is a phytochemical with promising effects on epilepsy treatment. However, its application has been hindered by its low bioavailability. In order to improve curcumin’s anti-seizure properties, increasing its bioavailability, here we proposed to micronize the compound through supercritical carbon dioxide processing, a suitable green chemistry technique to prepare and modify material properties. Here we investigated the anti-seizure potential of the classical antiepileptic drug valproate, curcumin in its natural state, and micronized curcumin in a PTZ-induced seizure model in zebrafish (*Danio rerio*). Concerning seizure development, valproate, curcumin and micronized curcumin showed protective effects, slowing seizure development both in larvae and adult animals. Nevertheless, considering the occurrence of the tonic-clonic seizure stage, only valproate and micronized curcumin reduced it, both in larvae and adult zebrafish, unlike non-processed curcumin. Our obtained results are very promising, since micronized curcumin showed effects that are similar to a classic antiepileptic drug, reducing seizure occurrence and slowing seizure progression.

## Introduction

Epilepsy is one of the most common neurological diseases, affecting around 65 million people worldwide^1^. The treatment is often pharmacological, and drugs act mostly through modulation of ion channels and neurotransmitter receptors^2^. Despite the high number of options, current antiepileptic drugs (AEDs) fail to suppress seizure occurrence in around 30% of epileptic patients^3,4^. Therefore, new drugs with different mechanisms of action may be required.

Curcumin is a polyphenolic phytochemical obtained from *Curcuma longa*. It is well known that curcumin inhibits the mammalian target of the rapamycin (mTOR) pathway^5^. mTOR inhibition has been proposed as a potential antiepileptogenic and anticonvulsive strategy in several epilepsy and seizure animal models^6–9^. Furthermore, curcumin inhibits the production of various pro-inflammatory cytokines that are significantly upregulated in several types of epilepsy. Considering that inflammatory pathways have pro-convulsant effects, curcumin’s anti-inflammatory properties are extremely significant in epilepsy^5^.

Curcumin has been extensively studied, and recently its promising therapeutic effect on epilepsy was evidenced^10–12^. This phytochemical did not prevent the occurrence of seizures, but weakened their severity and also showed a protective effect against cognitive deficits in a temporal lobe epilepsy model induced by *status epilepticus* in rats^10^. In addition, curcumin-loaded nanoparticles reduced memory deficits and cell loss in a pentylenetetrazole (PTZ)-induced kindling model^12^.

Nevertheless, despite its promising therapeutic effect on reducing comorbidities associated to epilepsy, curcumin’s anticonvulsant potential is still poor. Different studies have shown that curcumin’s effects are impaired by its low bioavailability^13,14^. Therefore, it is essential to investigate tools that aim to overcome this deficit.

**Figure 1:**
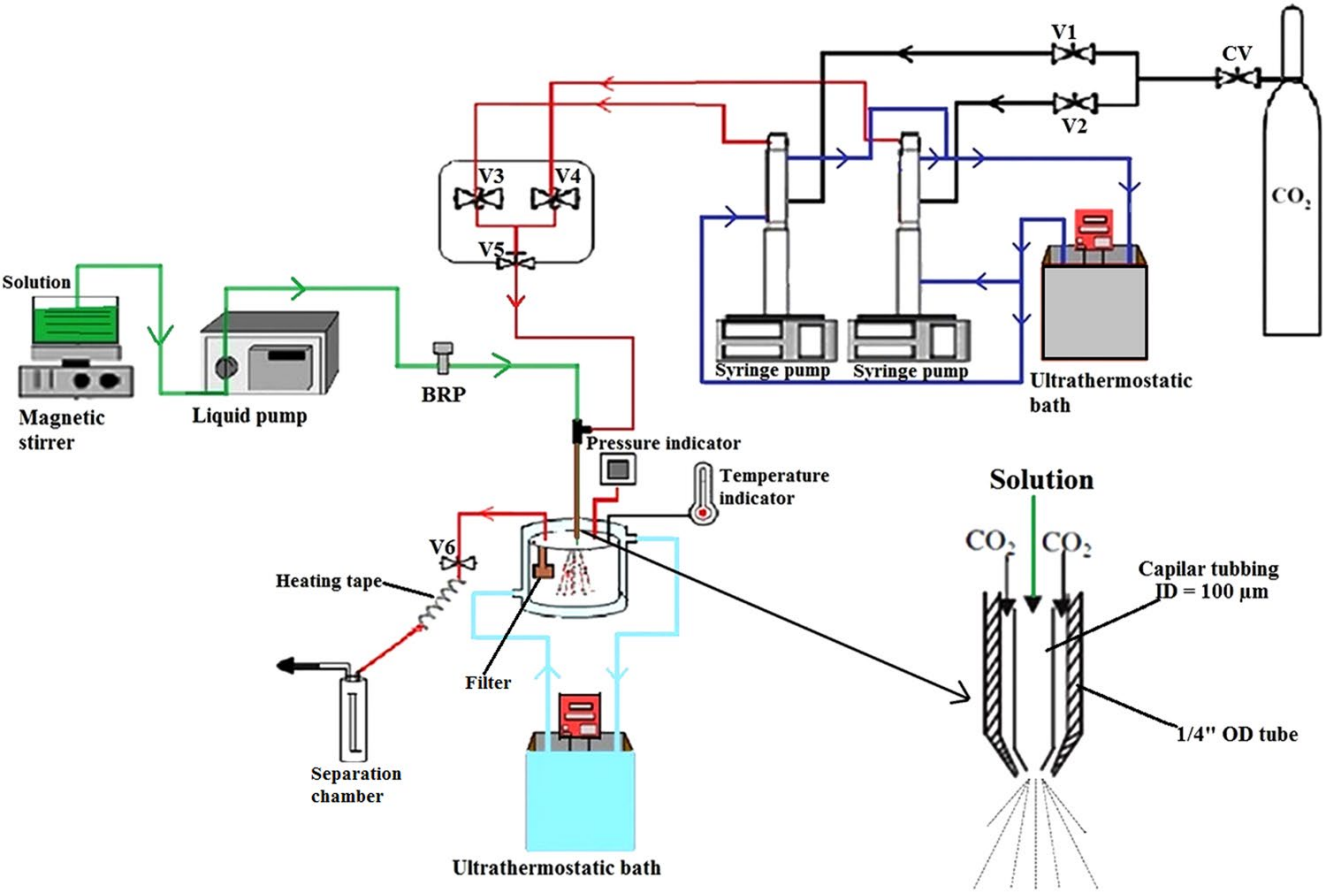
Schematic diagram of the experimental apparatus using the Solution Enhanced Dispersion by Supercritical Fluids technique. CV–Check–Valve; V1, V2, V3 and V4–Ball valve; V5 and V6–Needle valve; BRP–Back Pressure Regulator.

Drug bioavailability can be improved by micronizing the raw material by applying the supercritical carbon dioxide micronization technology^15,16^. Supercritical carbon dioxide is a suitable green chemistry technique to prepare and modify material properties (Fig. 1). This tool allows a greatly reduced particle size to be obtained, compared with the starting material, with a narrow size distribution, thus increasing its solubility and the specific surface contact area. Considering the potential of the micronization technology in reducing particle size and in increasing this narrow size distribution, solubility and surface contact area, we thought of applying it with the aim of improving the anticonvulsive potential of curcumin. Therefore, we investigated it in a consolidated seizure model.

Zebrafish is a small freshwater teleost that has been used as a model organism to study epileptic seizure occurrence, antiepileptic drug action, epilepsy-induced cognitive dysfunction, and alternative therapies for seizure control in epilepsy^8,17,18^. Pentylenetetrazole (PTZ)-induced seizures both in larvae and in adult zebrafish cause the behavioral and electrographic alterations that would be expected from a seizure episode^17,19^.

Since curcumin is a natural compound with a promising application in seizure control and its application nowadays has been hindered due to its low bioavailability, it is essential to develop tools that overcome this deficit and potentiate the effectiveness of curcumin in seizure control and in the treatment of epileptic patients. Here the micronization of the compound through supercritical carbon dioxide processing is proposed in order to improve curcumin bioavailability and consequently anti-seizure properties.

## Results

### Particle characterization

**Figure 2:**
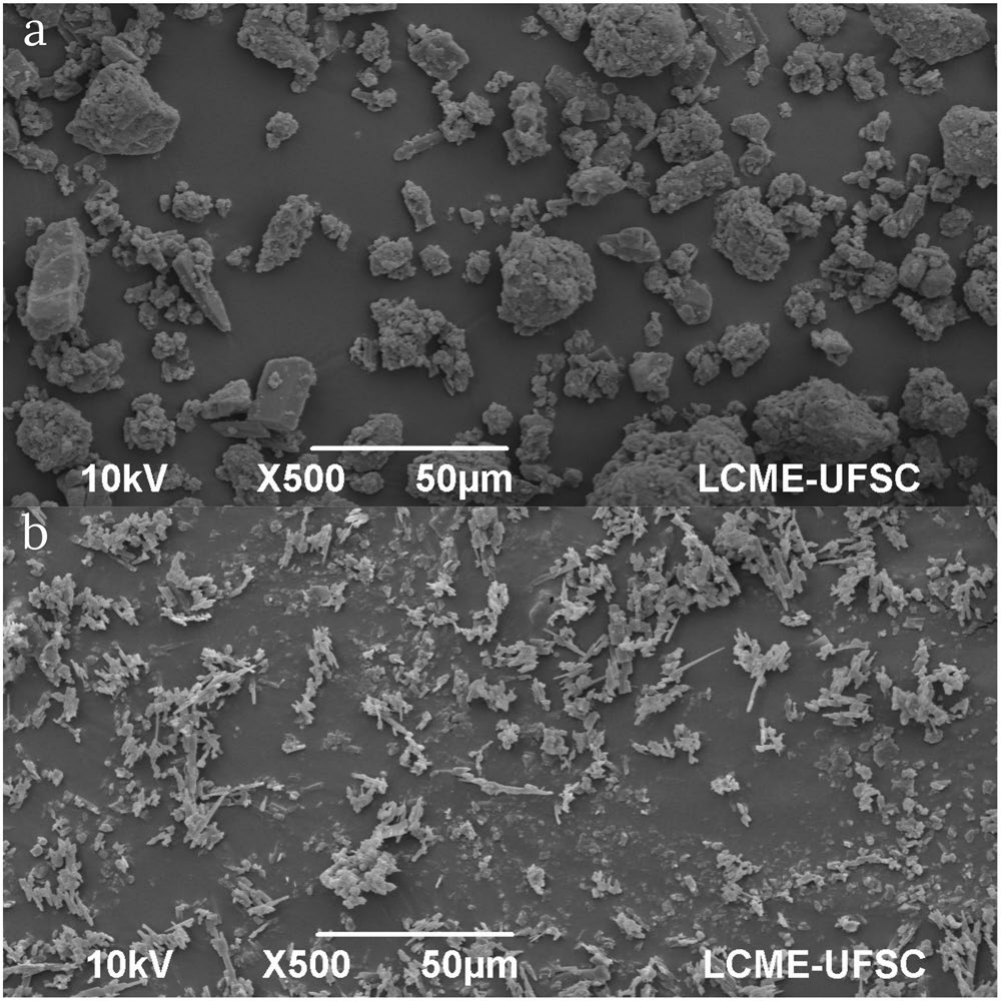
Scanning electron microscope of the produced particles.

Particle characterization and size values of non-micronized and micronized obtained compounds are presented in Table 1 and in Fig. 2.

**Table 1:** Particle size values of micronized and non-micronized (raw) compounds.

	D_min_ (µm)	D_max_ (µm)	D_p_ (µm)	VC (%)
Raw	3.81	33.56	12.40 ± 6.26	54.44
Micronized	0.69	8.51	2.35 ± 0.95	40.40

D_min_, D_max_ and D_p_ correspond to minimum diameter, maximum diameter, and average particle diameter respectively, while VC denotes the variation coefficient.

### Differential Scanning Calorimetry of the micronized and non-micronized samples

**Figure 3:**
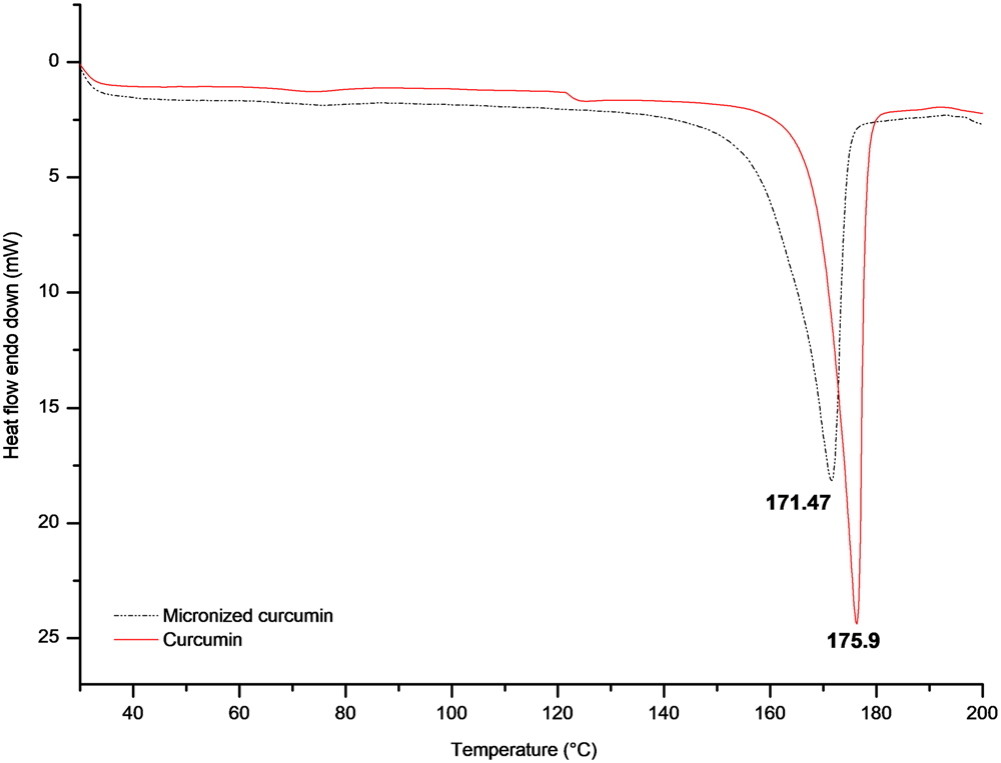
Differential scanning calorimetry of the micronized and non-micronized samples.

Differential Scanning Calorimetry (DSC) data (Fig. 3) showed that the detected curcumin melting point was 175.9 °C, while for micronized curcumin the melting point observed was 171.47 °C. Considering the ΔHf, it changed from 108 to 106 J·g^−1^ for curcumin and micronized curcumin respectively.

### Locomotor activity and exploratory behavior response following drug treatments

**Figure 4:**
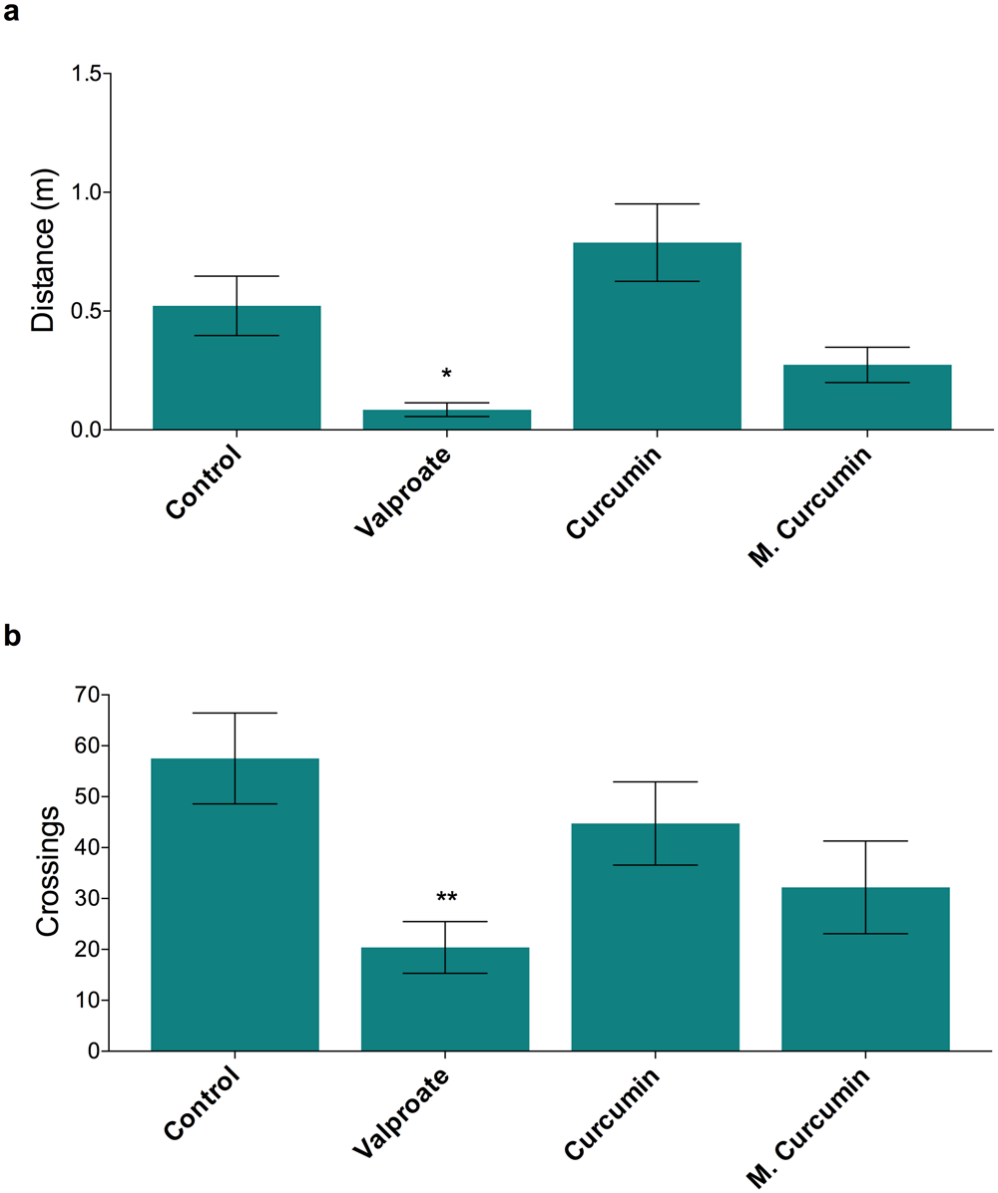
Effects of valproate, curcumin and micronized curcumin on behavioral parameters in zebrafish larvae: distance traveled (**a**) and line crossing (**b**) in the novel tank test. Data is expressed as mean ± S.E.M. One-way ANOVA followed by Dunnett’s post-test. n = 20. *P < 0.05, **P < 0.005 VS. control group. Zebrafish larvae were submitted to treatment with 0.1% DMSO (control group), curcumin and micronized curcumin at 1 μM and VPA at 3 mM.

**Figure 5:**
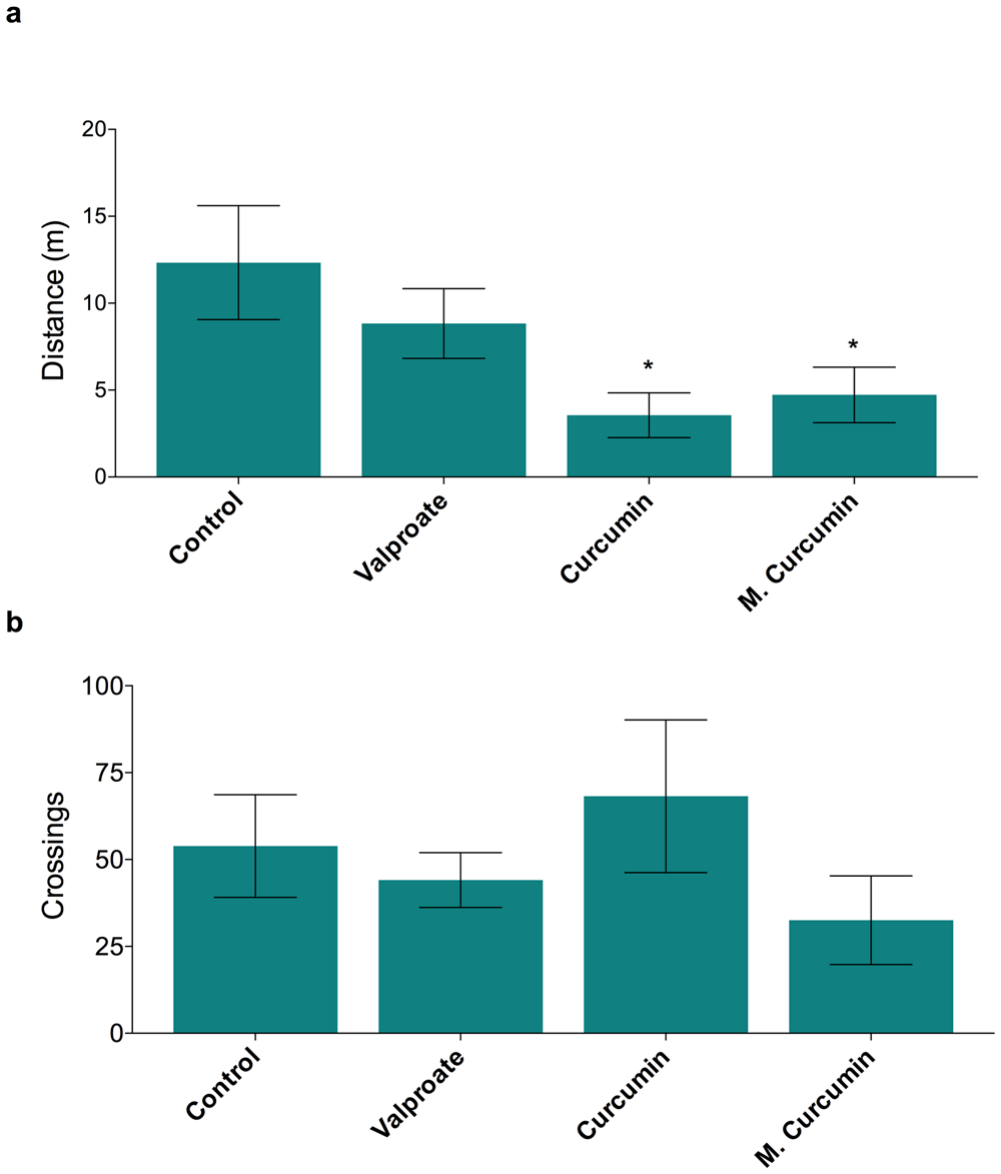
Effects of valproate, curcumin and micronized curcumin on behavioral parameters in adult zebrafish: distance traveled (**a**) and line crossing (**b**) in the novel tank test. Data is expressed as mean ± S.E.M. One-way ANOVA followed by Dunnett’s post-test. n = 18. *P < 0.05 VS. control group. Adult animals received the treatments of 1% DMSO (control group), curcumin and micronized curcumin at 0.50 mg/kg and VPA at 100 mg/kg.

In order to investigate the effects of curcumin and micronized curcumin on locomotor activity and exploratory behavior, animal behavior was analyzed on a novel tank apparatus during the 30 min following curcumin and micronized curcumin and prior to PTZ exposure. The same protocol was applied to verify the effects of valproate (VPA). Considering zebrafish larvae, obtained data showed that both curcumin and micronized curcumin did not induce alterations on distance traveled and line crossing, while VPA reduced both traveled distance (Fig. 4a; F(3,76) = 7.668; *P* = 0.0002) and the number of line crossings (Fig. 4b; F(3,76) = 4.012; *P* = 0.0105) compared with the control group. In adult zebrafish, curcumin and micronized curcumin reduced locomotion in comparison with non-treated animals (Fig. 5a; F(3,68) = 3.400; *P* = 0.0226). Finally, alterations on line crossing in adult animals were not detected (Fig. 5b).

### Seizure occurrence and development

**Figure 6:**
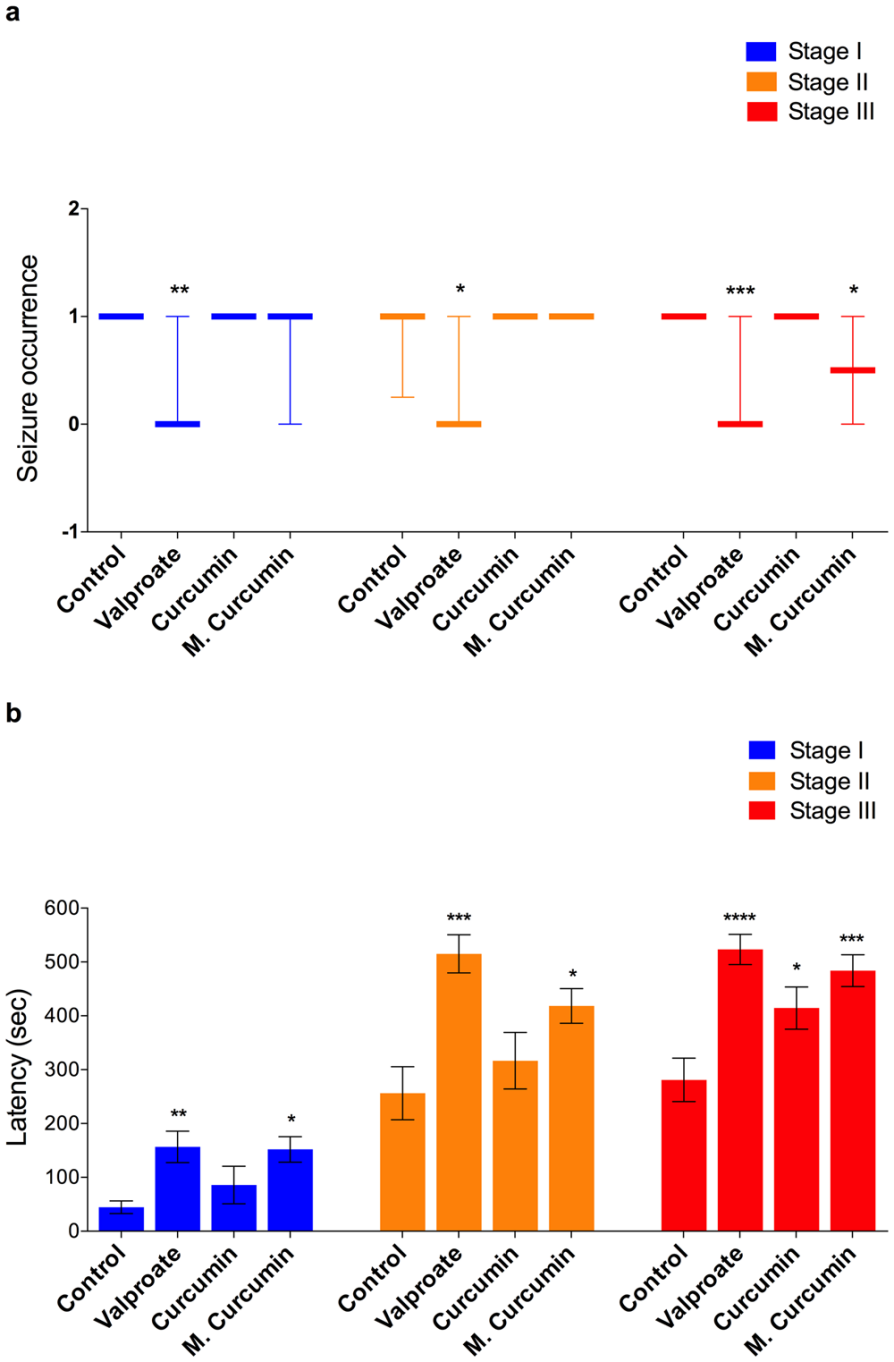
Effects of valproate, curcumin and micronized curcumin on the occurrence of each seizure stage (**a**) and latency to reach each seizure stage (**b**). (**a**) Data is expressed as a median with interquartile range. Kruskal-Wallis followed by Dunn’s test. n = 20. (**b**) Data is expressed as mean ± S.E.M. One-way ANOVA followed by Dunnett’s post-test. n = 20. *P < 0.05, **P < 0.005, ***P < 0.0005 and ***P < 0.0001 VS. control group in each seizure stage. Zebrafish larvae were submitted to treatment with 0.1% DMSO (control group), curcumin and micronized curcumin at 1 μM and VPA at 3 mM.

In zebrafish larvae, micronized curcumin showed ability to reduce seizure occurrence (Fig. 6a) and to slow seizure progression (Fig. 6b). Obtained data showed that VPA reduced the occurrence of seizure stages I (*P* = 0.0020) and II (*P* = 0.0024). Considering the occurrence of the tonic-clonic seizure stage (III), VPA and micronized curcumin reduced it (*P* = 0.0003). Larvae treated with VPA and micronized curcumin took longer to reach seizure stages I (F(3,76) = 4.261; *P* = 0.0078) and II (F(3,76) = 6.931; *P* = 0.0003). Finally, seizure stage III was slowed by VPA, curcumin and micronized curcumin (F(3,76) = 9.374; *P* < 0.0001).

**Figure 7:**
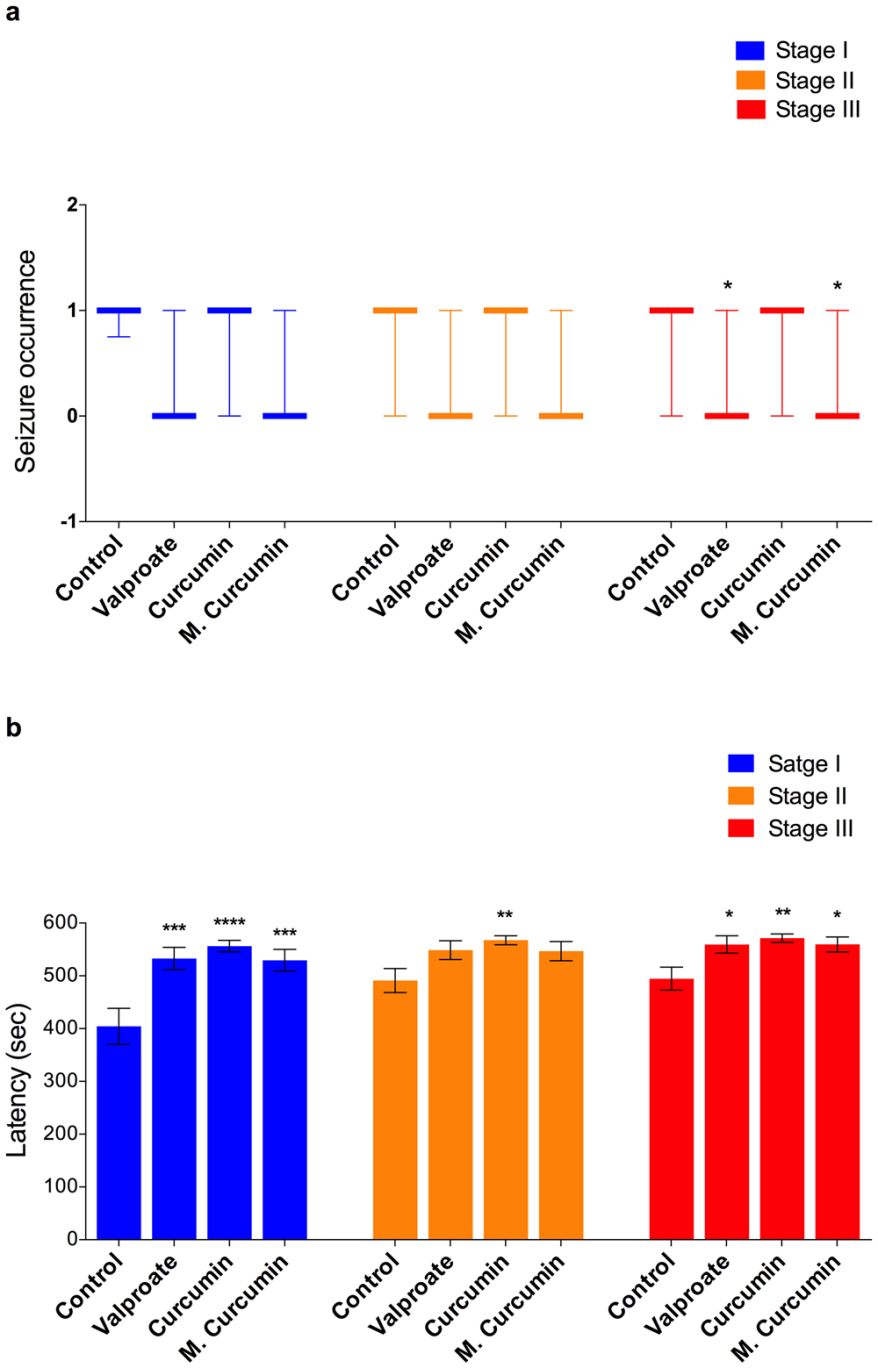
Effects of valproate, curcumin and micronized curcumin on the occurrence of each seizure stage (**a**) and latency to reach each seizure stage (**b**). (**a**) Data is expressed as a median with interquartile range. Kruskal-Wallis followed by Dunn’s test. n = 18. (**b**) Data is expressed as mean ± S.E.M. One-way ANOVA followed by Dunnett’s post-test. n = 18. Symbols indicate adjusted *P* value for each comparison. *P < 0.05, **P < 0.005, ***P < 0.0005 and ***P < 0.0001 VS. control group in each seizure stage. Adult animals received the treatments of 1% DMSO (control group), curcumin and micronized curcumin at 0.50 mg/kg and VPA at 100 mg/kg.

Micronized curcumin also provided a protective effect in adult zebrafish. Micronized curcumin and VPA reduced the occurrence of seizure stage III (Fig. 7a; *P* = 0.0161). Considering seizure progression, both curcumin and micronized curcumin provided a protective effect (Fig. 7b). Seizure stage I was slowed by VPA, curcumin and micronized curcumin (F(3,68) = 8.711; *P* < 0.0001). Seizure stage II was slowed by curcumin (F(3,68) = 3.502; *P* = 0.0200). Finally, seizure stage III was slowed by VPA, curcumin and micronized curcumin (F(3,68) = 4.817; *P* = 0.0042).

## Discussion

Our obtained results are very promising, since micronized curcumin showed effects that are similar to a classic AED. Thirty minutes after its administration, micronized curcumin shows an anticonvulsive effect corresponding to VPA both in larval and in adult zebrafish. Regarding seizure development, valproate, curcumin and micronized curcumin showed protective effects, slowing seizure development both in larvae and adult animals. Nevertheless, considering the occurrence of the tonic-clonic seizure stage, only valproate and micronized curcumin reduced it, both in larvae and adult zebrafish, unlike non-processed curcumin.

In addition, we highlight that micronized curcumin was administered in a much smaller amount than VPA. It is worth noting that micronized curcumin was administered in a concentration 3.000 times smaller for larvae and in a dose 200 times smaller for adults. Such low drug dosages are extremely significant for human health. Additionally, curcumin is a spice commonly consumed by the population, already being present in people’s diets^20^.

The DSC results shown in Fig. 3 demonstrated that the melting point changed from 175.9 °C (raw curcumin) to 171.47 °C (micronized curcumin), which indicates the modification of the crystalline structure as it causes changes in the melting point of the compound, such as dissolution, among other properties^21,22^. Another important fact to be highlighted is that the ΔHf of the compound changed from 108 to 106 J·g^−1^. According to a previous study that investigated the micronization of curcumin with the Solution Enhanced Dispersion by Supercritical Fluids (SEDS) technique (the same used in this work), the reduction in the ΔHf can be attributed to the disrupted molecule chains and also to the extent of decreased crystallization caused by the SEDS process^23^.

Changes in the melting point were also observed by Aguiar *et al*. when studying n-acetylcysteine (NAC) micronization with the SEDS technique^15^. In that work, *in vivo* evaluation of the micronized versus non-micronized compounds showed that micronized NAC exhibited a similar biological activity even at a concentration 100-fold lower than the non-micronized NAC. The authors concluded that micronization with the SEDS technique increased the dissolution rate, improved antioxidant activity and altered the crystalline structure of the compound and its melting point, which led to significant improvements in both *in vivo* and *in vitro* tests^15^.

Behavioral alteration is a common effect of AEDs. The obtained data showed that curcumin and micronized curcumin reduced the locomotion and exploratory behavior of adult animals. Nevertheless, curcumin and micronized curcumin did not induce behavioral alterations in larval zebrafish, while the VPA treatment provoked decreases in both locomotion and exploratory activity. Neurodevelopmental comorbidities could be extremely disabling. The administration of AEDs during the neurodevelopmental stage could provoke behavioral changes and be detrimental to brain development^24^. Our results showed that the acute treatment with curcumin and micronized curcumin did not induce behavioral alterations in zebrafish larvae, which is very positive. More studies are necessary to investigate their chronic and late effects.

Finally, another relevant point of the present research must also be highlighted. Curcumin micronization was developed by a “green technique”, which does not produce any environmental impacts, since it does not comprise residue generation^15,16^.

In a broad sense, the present report makes an important advance in epilepsy treatment, considering that new drugs with different mechanisms of action are necessary. Curcumin acts inhibiting the mTOR pathway and inflammatory mechanisms, which are related to epilepsy^5,11^. In accordance, different studies have shown neuroprotective effects of this phytochemical in animal models of epilepsy^10–12^.

In this context, curcumin is clearly beneficial for the treatment of epilepsy but surprisingly it has not been used to date due merely to its low bioavailability, which mainly impairs its anticonvulsant action. Now, this study presents the solution to this problem. Our results show that the micronization of curcumin improves its efficacy. As a matter of fact, 30 min after its administration, micronized curcumin shows an anticonvulsive effect that corresponds to a classic AED.

## Methods

### Animals

Adult wild-type zebrafish (*Danio rerio*) of both sexes were obtained from a local supplier and acclimated for 4 weeks before the experiments were conducted at the bioterium at Universidade Comunitária da Região de Chapecó, Brazil. Zebrafish embryos were obtained from natural mating of adult zebrafish maintained in bred tanks. Fertilized eggs were collected, washed with system water (reverse osmosis water equilibrated with Instant Ocean salts), freed of debris, and transferred to sterile cell culture plates. Plates were maintained in an incubator at 28.5 °C and monitored daily until 7 dpf. Adult animals were housed in thermostated thanks filled with unchlorinated water constantly aerated at a targeted temperature of 26 ± 2 °C. Fish were kept under a 14–10 h light/dark cycle photoperiod and fed daily, three times a day, with fish food that was supplemented with live artemia. Euthanasia of animals was performed after the tests and was practiced by immersion in a tricaine solution. The protocol was approved by the Ethics Committee for Animal Use (CEUA) of Unochapecó (Protocol #014/2016) and followed the guidelines of Conselho Nacional de Controle de Experimentacão Animal (CONCEA).

### Materials

Curcumin, pentylenetetrazole (PTZ), tricaine and valproate (VPA) were purchased from Sigma Aldrich (St. Louis, MO, USA).

### Curcumin Micronization with the Solution Enhanced Dispersion by Supercritical Fluids Technique

The Solution Enhanced Dispersion by Supercritical Fluids technique *(SEDS)*, experimental equipment and procedure used to micronize curcumin with supercritical CO_2_ as anti-solvent, is described in detail by previous studies^25,26^. The process parameters adopted in the present report were based on optimized trans-resveratrol and N-acetylcysteine micronization data provided by Aguiar *et al*.: solute concentration of 20 mg∙mL^−1^, temperature at 35 °C, anti-solvent flow rate of 20 mL∙min^−1^, solution flow rate of 1 mL∙min^−1^ and operating pressure of 8 bar^15,27^.

### Particle Characterization

In order to characterize the particles, the following analyzes were performed: Morphology by Scanning Electron Microscopy (SEM) and determination of particle size by the software Meter Size (version 1.1)^15^.

### Differential scanning calorimetry

The melting point of the compound was determined using a system of differential scanning calorimetry (Jade-DSC, Perkin Elmer). The samples (5–10 mg) were prepared in an aluminum pan, and DSC measurements were performed by heating at 30 to 200 °C at a rate of 10 °C/min in an inert atmosphere (N2 flow: 10 mL·min^−1^)^15^.

### Treatments

All animals received the respective treatment 30 min before the PTZ exposure. In order to verify the zebrafish response to a classic AED in our PTZ-induced seizure model, we treated a group of animals with VPA. All groups were treated and analyzed in an identical manner. Curcumin and VPA concentrations and doses were selected based on preliminary studies conducted in our laboratory.

Zebrafish larvae (n = 20) were individually placed in 6 well plates (1 larva per well with 5000 μL of solution) and submitted to treatment with 0.1% DMSO (control group), curcumin at 1 μM, micronized curcumin at 1 μM or VPA at 3 mM for 30 min. In adult animals, the 1% DMSO (control group), curcumin at 0.50 mg/kg, micronized curcumin at 0.50 mg/kg and VPA at 100 mg/kg were applied by intraperitoneal (i.p.) injection. I.p. injections were given using a 3/10-ml U-100 BD Ultra-FineTM Short Insulin Syringe 8 mm (5/16) × 31 G Short Needle (Becton Dickin-son and Company, New Jersey, USA) according to the protocol established by Phelps *et al*.^28^. Anesthesia of adult animals prior to the injection was given by their immersion in a 10% tricaine solution until the animals showed lack of motor coordination and reduced respiration rate. After the injection, the animals were placed in a separate tank with aerated unchlorinated tap water (26 ± 2 °C) to recovery from anesthesia.

### Locomotor and exploratory activity

In order to analyze the effects of curcumin and micronized curcumin on locomotor and exploratory activity, all animals were individually submitted to the novel tank test^29,30^. Animal behavior was recorded during the 30 min following curcumin and micronized curcumin and prior to PTZ exposure. The same protocol was applied to verify the effects of VPA. Larvae were individually placed in 6 well plates (1 per well, with 5000 μL of solution). Adult animals were individually placed in glass tanks (12 cm × 8 cm × 13.5 cm, length × width × height). The animal behavior was registered by a video camera for 30 min and further analyzed using the ANY-Maze® recording software (Stoelting Co., Wood Dale, IL, USA) to track the locomotor and exploratory behavior of the animals. To analyze the behavior, the larvae novel tank apparatus was divided into center and periphery. The adult novel tank apparatus was divided into bottom, middle and upper. Distance traveled and zone line crossing were analyzed.

### PTZ-induced seizures

To induce seizures, zebrafish were individually exposed to 3 mM PTZ^19^. Larvae were individually exposed to PTZ in 6-well plates. PTZ solution was applied in the larval medium in order to obtain a final concentration of 3 mM in a total volume of 5000 μl. Adult zebrafish were individually exposed to 3 mM PTZ by their immersion in 250 mL beakers. All PTZ treatments were videotaped and evaluated later by trained observers. The seizure-like behavior was classified according to each stage: stage I—dramatically increased swimming activity, stage II—whirlpool swimming behavior, and stage III—clonus-like seizures followed by loss of posture, when the animal falls to one side and remains immobile for 1–3 s, as previously reported for zebrafish^19^. The animals were submitted to the PTZ treatment until they reached stage III, which corresponds to the tonic–clonic seizure stage in zebrafish, or until 600 s.

The occurrence of each seizure stage and the latency to the first behavioral sign of each seizure stage were analyzed. Experiments were performed in triplicate, on different days. Experimental groups consisted of 20 zebrafish larvae and 18 adult zebrafish, in an equal number of males and females. All tests were done in the morning.

### Statistical analysis

Normality of the data distribution was confirmed with the Kolmogorov-Smirnov test. Obtained data about the occurrence of each seizure stage was analyzed using Kruskal-Wallis followed by Dunn’s test, and results are expressed as a median with interquatile range. Considering the latency to reach each seizure stage and behavioral parameters, data was analyzed using One-way (ANOVA) followed by Dunnett’s post-test. These results are expressed as a mean ± standard error of the mean (S.E.M.).
